# Are Video Games a Gateway to Gambling? A Longitudinal Study Based on a Representative Norwegian Sample

**DOI:** 10.1007/s10899-018-9781-z

**Published:** 2018-06-05

**Authors:** Helge Molde, Bjørn Holmøy, Aleksander Garvik Merkesdal, Torbjørn Torsheim, Rune Aune Mentzoni, Daniel Hanns, Dominic Sagoe, Ståle Pallesen

**Affiliations:** 1 Department of Clinical Psychology, University of Bergen, Bergen, Norway; 2 Department of Psychosocial Science, University of Bergen, Bergen, Norway; 3 Hochschule Darmstadt, University of Applied Sciences, Darmstadt, Germany

**Keywords:** Gambling, Video gaming, Longitudinal, Representative sample, Cross-lagged

## Abstract

The scope and variety of video games and monetary gambling opportunities are expanding rapidly. In many ways, these forms of entertainment are converging on digital and online video games and gambling sites. However, little is known about the relationship between video gaming and gambling. The present study explored the possibility of a directional relationship between measures of problem gaming and problem gambling, while also controlling for the influence of sex and age. In contrast to most previous investigations which are based on cross-sectional designs and non-representative samples, the present study utilized a longitudinal design conducted over 2 years (2013, 2015) and comprising 4601 participants (males 47.2%, age range 16–74) drawn from a random sample from the general population. Video gaming and gambling were assessed using the Gaming Addiction Scale for Adolescents and the Canadian Problem Gambling Index, respectively. Using an autoregressive cross-lagged structural equation model, we found a positive relationship between scores on problematic gaming and later scores on problematic gambling, whereas we found no evidence of the reverse relationship. Hence, video gaming problems appear to be a gateway behavior to problematic gambling behavior. In future research, one should continue to monitor the possible reciprocal behavioral influences between gambling and video gaming.

## Introduction

Behavioral addictions can be understood as the excessive and continued involvement in activities that cause harm to the addict or to persons with important relationships to the addict (American Psychiatric Association 2013). The conceptualization of such addictions is often modeled on the diagnostic criteria for substance abuse disorders. Currently, the most researched and so far only behavioral addiction recognized by formal psychiatric nosology, is gambling disorder (Petry et al. 2014). The term gambling generally refers to games where the player bets money or other valuable items on an outcome that fully or partly relies on chance (Hodgins et al. 2011). Historically, gambling can be considered an almost universal feature of human societies. However, the diversity and accessibility of formalized gambling activities and platforms have increased dramatically during the last decades. The increased accessibility has entailed the development of massive commercial casinos and gambling destinations as well as the rising availability of online gambling. Likewise, the video gaming industry has expanded considerably during the past decade, similarly to the development of the gambling industry (King et al. 2010, 2015).

For most people who gamble occasionally, gambling primarily represents recreation and an enjoyable activity (Back et al. 2011). However, some people develop problematic patterns of gambling activity that are harmful to themselves and to society. Common characteristics of problematic gambling patterns include inappropriate amounts of time and money spent on gambling activities and the continuation of gambling activities even though gamblers’ personal relationships, financial assets, status or other important aspects of life are significantly impaired. Excessive gambling is thus associated with debts, bankruptcy and professional problems, as well as substantial psychological distress (Nower et al. 2015; Kessler et al. 2008). It is estimated that each problem gambler affects between five to fifteen other persons (Kalischuk et al. 2006). The estimated prevalence rates of gambling disorder vary depending on the specific country, survey year, and method of measurement; however, according to a standardized past year rate of problem gambling it ranges from 0.5 to 7.6%, with the average rate across all countries is 2.3% (Williams et al. 2012). Gambling problems are more common among some subsets of the general population, such as younger people, male gender, single or divorced/separated marital status, and low socioeconomic status (Toneatto and Nguyen 2007; Petry et al. 2005). Gambling disorder is also associated with increased risk of other addictive disorders, especially alcoholism and substance abuse disorder (Petry 2005). Other mental illnesses, such as mood disorders and anxiety disorders, have further been shown to have high comorbidity with gambling disorder and comorbid mental illnesses might predispose high-risk individuals to develop or worsen pre-existing gambling problems (Kessler et al. 2008; Dowling et al. 2015).

Nevertheless, little is known about the relationship between measures of problematic gambling and video gaming especially in terms of temporal associations. It is important, however, to elucidate the latter relationship for several reasons. Gaming and gambling seem increasingly to be converging. Gambling is constantly being digitized and diversified into a multitude of online games at the same time as video games increasingly contain themes and elements from more traditional gambling activities. Furthermore, websites offering both gaming and gambling increase dual accessibility of the activities, which may cause higher levels of involvement (Fisher and Griffiths 1995). New developments such as betting on e-sport represent further examples of the increasing intertwinedness of gaming and gambling (Hutchins 2008).

While there is concern regarding how to properly distinguish gaming and gambling (King et al. 2015), the potential for comorbidity and “recruitment” from one problem category to the other is immanent. So far, there are mixed results regarding comorbidity between video gaming and gambling. In a recent study with a sample of Australian video gamers, no significant relationship between frequency of video gaming and gambling was found (Forrest et al. 2016). However, some studies suggest a link between involvement in gaming and gambling. For example, the frequency of visits to a non-gambling video game arcade was found to be positively associated with the frequency of disordered gambling (Ladouceur and Dubé 1995). A study of children found a positive association between time spent playing video games and the likelihood to partake in risk-taking gambling (Gupta and Derevensky 1996). A study of German students aged 12–25 (Walther et al. 2012) reported a significant correlation between problematic video gaming and gambling. A recent study by McBride and Derevensky (2016) examined the gambling and video game behaviors of 1229 adolescents and young adults. The results indicate that gamblers, compared with non-gamblers, were more likely to play video games and that video gamers, compared to non-gamers, were more likely to take part in gambling activities. Against this backdrop, the present study seeks to explore the directionality between problematic video gaming and gambling using a representative population sample and a longitudinal design based on assessments separated by 2 years.

## Method

### Procedure

We conducted a longitudinal panel study covering a 2-year period. The first wave was carried out in 2013, the second wave in 2015. The survey was conducted on behalf of the Norwegian Gaming and Foundation Authority and was approved by the Regional Committee for Medical and Health Related Ethics in Western Norway (REK-Vest, project no. 2013/120). In all, 24,000 people aged 16–74 were randomly selected from the National Population Registry of Norway and invited to participate in a postal survey in 2013. A letter attached to the survey informed that all respondents would automatically be included in a raffle with a chance to win a gift voucher worth NOK 500. A maximum of two reminders were sent to those who had not responded to the survey. This same procedure was used during the second wave.

### Participants

In all, 10,081 valid answers were collected during the first wave (response rate 43.6%). These respondents were invited to participate again in 2015. In the second wave, 5809 valid answers were collected (response rate 57.6%); hence, these 5809 individuals make up the basis of this panel study. All participants included in the present study confirmed in at least one of the two waves that they had played video games during the previous 6 months and/or had participated in gambling activities during the past 12 months. This amounted to 4601 respondents, 2172 (47.2%) males and 2429 (52.8%) females, with an age range in wave 1 of 16–74 years of age (mean = 48 years, SD = 15.1).

### Instruments

The Gaming Addiction Scale for Adolescents (GASA) is a validated and frequently used measurement instrument. The GASA is based on previous research on video gaming and the biopsychosocial model of addiction (Griffiths 2005; Lemmens et al. 2009). Respondents are asked to respond to items reflecting different aspects of problematic video gaming during the previous 6 months. The response options range from *never* (0) to *very often* (4). The short version of this questionnaire contains seven items, each tapping into one of the criteria of addiction (salience, tolerance, mood modification, relapse, withdrawal, conflict and problems). We used a validated Norwegian version (Mentzoni et al. 2011). In the present study, the Cronbach’s alpha was 0.83 in both waves. A composite sum score was computed for each individual participant by adding the scores on the items in the scale. Video gaming behavior was regarded as a phenomenon with a continuous severity dimension, where higher composite scores on the GASA indicate higher involvement/severity.

To assess the extent of gambling problems, we administered the Canadian Problem Gambling Index (CPGI; Ferris and Wynne 2001). The CPGI consists of nine items, five of which measure problematic gambling behavior, and four measure negative consequences of gambling behavior. Each item is answered on a 4-point scale ranging from *never* (0) to *always* (3). Cronbach’s alpha was 0.92 in wave 1 and 0.84 in wave 2. A composite score was computed by adding the scores on the items in the scale for each individual participant. Gambling behavior was regarded as a phenomenon with a continuous severity dimension, where higher ordinary sum scores indicate higher involvement/severity.

### Analyses

To assess the unidimensionality of the CPGI and the GASA, we applied principal component analysis. The correlations among the seven items of the GASA and the nine items of the CPGI indicated that they each measure one underlying construct. We could therefore use the composite scores for both measures when conducting our main statistical analyses.

We used structural equation modeling (SEM) for the main analyses. To explore the correlations over time, we applied an autoregressive cross-lagged panel model using the composite scores (i.e., a model without indicators) for both video gaming and gambling (see Lemmens et al. 2011b, for a comparable model). The lavaan package for structural equation modelling in R (Rosseel 2011) was used to fit both models. Full information maximum likelihood (FIML) was applied, where the missing values were estimated from all available data. Robust Huber–White standard errors were applied due to skewness within the data. Because outliers could affect the correlations, we ran a model without outliers to investigate whether this was the case. The results revealed that the directional associations remained the same, and only marginal differences in strength appeared. Based on these results, we decided to use all data in our analyses.

## Results

### Descriptive and Correlational Statistics

Descriptive data for the GASA and the CPGI are provided in Table 1. In the two waves, 3593 and 3533 respondents, respectively, confirmed that they had participated in gambling activities during the prior 12 months, and thus completed the CPGI. The numbers of respondents who confirmed that they had played video games during the past 6 months were 1719 in wave 1 and 1781 in wave 2.

**Table 1:** Descriptive statistics of GASA and CPGI in wave 1 and wave 2

Variable	N	Range	Minimum	Maximum	Mean (SD)	Median
GASA *W*1	1719	23	0	23	2.56 (3.60)	1.00
GASA *W*2	1781	28	0	28	2.33 (3.52)	1.00
CPGI *W*1	3593	1	0	1	0.14 (0.35)	0.00
CPGI *W*2	3553	1	0	1	0.14 (0.34)	0.00

In both waves, respondents generally had low scores on both the GASA and the CPGI, as indicated by the means. The means and standard deviations differed little between the first and the second wave, suggesting little change at the group level in terms of gaming and gambling problems. The variance was also similar across the two waves for both measures.

Significant correlations were found between all variables, where the strongest correlations were between the wave 1 and wave 2 measures of the same construct. The largest coefficient was found for GASA in wave 1 and wave 2 (*r *= 0.60). The correlation coefficient for CPGI scores in wave 1 and wave 2 was *r *= 0.44. For more details, see Table 2.

**Table 2:** Correlation coefficients within and between the study variables in wave 1 and wave 2

	GASA *W*1	GASA *W*2	CPGI *W*1	CPGI *W*2	Sex	Age
GASA *W*1	–					
GASA *W*2	0.60**	–				
CPGI *W*1	0.25**	0.19**	–			
CPGI *W*2	0.25**	0.19**	0.44**	–		
Sex	0.05**	0.06**	0.09**	0.09**	–	
Age	− 0.28**	− 0.30**	− 0.07**	− 0.10**	0.09**	–

Sex was coded; female = 0, male = 1

*p <0.05; **p <0.01

### Autoregressive Cross-Lagged Panel Analysis

**Fig. 1:**
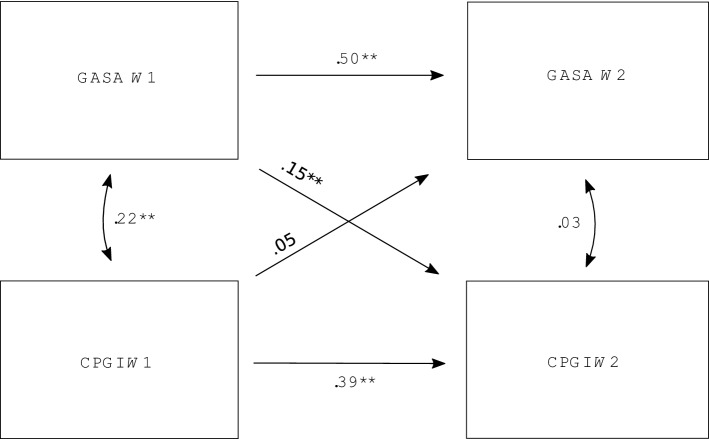
Configural model of the cross-lagged panel analysis. *p < 0.05; **p < 0.01

The results are displayed in Table 3 and Fig. 1. A strong model fit was indicated by the root mean square error of approximation (RMSEA = 0.000) and Tucker-Lewis Index (TLI = 1.000). This strong model fit is due to the use of observed composite scores with no indicators. The analysis showed that the measures of gambling and video gaming problems had a statistically significant positive covariation (*β* = 0.22, *p *<0.01) in wave 1. In wave 2, no such relationship was found (*β *= 0.03, *p *<0.36). As shown by the horizontal line in Fig. 1, scores on the GASA in wave 1 significantly predicted scores on the GASA in wave 2 (*β *= 0.50, *p *< 0.01). CPGI scores in wave 1 also significantly predicted CPGI scores in wave 2 (*β *= 0.39, *p* < 0.01).

**Table 3:** Autoregressive cross-lagged panel model: covariances, regression weights and model fit indices

Paths	Unstandardized estimates	Standardized estimates
Covariances		
GASA *W*1 ↔ CPGI *W*1	0.26**	0.22
GASA *W*2 ↔ CPGI *W*2	0.03	0.03
Sex ↔ age	0.64**	0.08
Autoregressive paths		
GASA *W*1 → GASA *W*2	0.49**	0.50
CPGI *W*1 → CPGI *W*2	0.39**	0.39
Cross-lagged paths		
GASA *W*I → CPGI *W*2	0.02**	0.15
CPGI *W*1 → GASA *W*2	0.45	0.05
Control variables		
Sex → GASA *W*1	0.54**	0.08
Sex → GASA *W*2	0.07	0.01
Sex → CPGI *W*1	0.07**	0.10
Sex → CPGI *W*2	0.04**	0.06
Age → GASA *W*1	− 0.07**	− 0.29
Age → GASA *W*2	− 0.03**	− 0.14
Age → CPGI *W*1	− 0.00 **	− 0.09
Age → CPGI *W*2	− 0.00**	− 0.05

Sex was coded; female = 0, male = 1

*p <0.05; **p <0.01

In addition, we investigated the effects of sex and age on the GASA and CPGI. Analysis revealed that men reported higher total scores on the GASA compared to women in wave 1 (*β *= 0.08, *p *< 0.01); at the second wave, this relationship was no longer significant (*β *= 0.01, *p *< 0.65). Men reported higher scores on the CPGI compared to women in both waves (wave 1: *β *= 0.10, *p* < 0.01; wave 2: *β *= 0.06, *p* < 0.01). Age was found to be significantly and negatively related to the GASA scores in both waves (wave 1: *β *= − 0.29, *p *< 0.01; wave 2: *β* = − 0.14, *p *< 0.01). The same tendency, albeit smaller, was found for scores on the CPGI in both waves (wave 1: *β *= − 0.09, *p *= 0.01; wave 2: *β *= − 0.05, *p *< 0.05). Hence, the results indicate that younger respondents reported higher scores on both the GASA and the CPGI measures in both waves.

The cross-lagged analyses also revealed that the scores on the GASA in wave 1 significantly predicted scores on the CPGI in wave 2 (*β *= 0.15, *p* < 0.01). No such relationship was found between scores derived from the CPGI in wave 1 and scores on the GASA in wave 2 (*β* = 0.05, *p* < 0.12). This shows that a measure of video gaming problems is positively related to a measure of gambling problems at a later time, while the reverse relationship is not statistically significant. Estimates of intercepts and variance for the variables are provided in Table 4. They show higher scores on both measures in wave 1.

**Table 4:** Autoregressive cross-lagged panel model: intercepts and variance

	Unstandardized estimates	SE	Standardized estimates
Intercepts			
GASA *W*1	4.45**	0.33	1.26
GASA *W*2	2.21**	0.35	0.65
CPGI *W*1	0.15**	0.03	0.42
CPGI *W*2	0.06	0.03	0.16
Sex	1.47**	0.01	2.95
Age	50.00**	0.22	3.30
Variance			
GASA *W*1	11.33**	0.66	0.92
GASA *W*2	7.99**	0.66	0.68
CPGI *W*1	0.12**	0.00	0.99
CPGI *W*2	0.09**	0.00	0.78
Sex	0.25**	0.00	1.00
Age	229.57**	3.74	1.00

Sex was coded; female = 0, male = 1

**p < 0.01

## Discussion

A finding consistent with the research literature in general is that there were positive correlations between measures of the same construct over time, suggesting some stability over the 2-year period (Williams et al. 2015).

As for the relationship between gaming and gambling, we found a significant positive correlation between a score on the gaming problem scale and gambling at the first wave. However, this was not the case in the second wave, after controlling for the influence of sex and age. This indicates an inconsistency over time in the relationship between gambling and video gaming, which seems to be plausible given the mixed results on the strength of this relationship and the somewhat unstable and transitional nature of gambling and video gaming problems (LaPlante et al. 2008; Kuss et al. 2014; Thege et al. 2015). Another possible and perhaps complementary explanation for this finding may be that our sample had aged 2 years, which may have further reduced an initially weak correlation. This result may be seen in light of one of the most consistent findings in video game research—namely, that younger age is strongly correlated with more frequent playing (Ferguson et al. 2011; Toneatto and Nguyen, 2007).

Perhaps most interestingly, our results show that scores on the gaming problem scale at wave 1 predicted the scores on the gambling problem scale at wave 2, but there was no evidence of the reverse relationship. This suggests that video gaming constituted a risk factor for gambling 2 years later. Studies exploring the potential causal relationship between video gaming and gambling based on longitudinal designs and representative samples are very rare. The few existing studies generally show evidence of increased gambling among high-frequency video gamers (McBride and Derevensky 2016). However, there are mixed results regarding the strength of this relationship and whether extraneous variables are likely to mediate heightened involvement in both gaming and gambling (Delfabbro et al. 2009). Results from the current study suggest and support a direct and causal relationship between video gaming and gambling. One possible explanation for the relationship between problematic gaming and gambling is that the participants had become 2 years older. This could indicate that a significant subset of those involved with gaming developed an involvement with gambling during the transition. As age restrictions apply more strictly and widely to gambling activities than to video gaming, most people who eventually start to gamble are introduced to video games at an earlier age. It is thus conceivable that those who follow a path progressing from problematic gaming to gambling are generally prone to addictive behaviors and that the manifestation is moderated by age (Sussman et al. 2011). Another set of factors possibly augmenting such a trajectory is the increasing convergence between video gaming and gambling (King et al. 2010). This trend manifests itself, for example, in an increasing number of gambling products that adopt features from video games and also more video games containing intrinsic gambling themes (King et al. 2012, 2014; Walther et al. 2012). Furthermore, advertisements and game-related incentives are substantial for enticing players to games that involve monetary stakes and outcomes (King et al. 2015; Gainsbury et al. 2014b). In addition, formal and informal gambling has become a large part of e-sports (Holden et al. 2016).

It is thus conceivable that all these factors—age related transitions, proneness to addictive behavior, digital convergence and strategic marketing—may explain why this particular directionality was found. Some previous studies indicate that factors such as physical proximity of video gaming and gambling opportunities could lead to an increased number of problem gamblers, and a similar type of exposure effect could potentially operate in the world of digital and online games (Fisher and Griffiths 1995; Gainsbury et al. 2014a; Ladouceur and Dubé 1995). Keeping in mind that such a particular pathway is unlikely to apply to the majority of problem gamblers, it would still be very informative for research, clinical practice, and prevention agencies to be aware of the possibility that subgroups of gamers may be prone to developing gambling problems in line with the mechanisms/pathways outlined here.

Being male predicted higher scores on the CPGI in both waves. The same was found for GASA in the first wave, which could be expected from previous investigations (Petry et al. 2015; Rehbein et al. 2016). This was not the case in the second wave, as gender was found to be unrelated for GASA. The interpretation of this latter finding is that there are no gender differences beyond the association controlled for in wave 1. The finding of male preponderance and increased involvement in gambling and/or gaming are in accordance with previous research (Pallesen et al. 2016; Wittek et al. 2016). Still, attitudes toward video gaming activities seem to be changing, possibly due to more widespread acceptance, less restrictive stereotypes of gamers and increased interest on the part of females in video games (Ipsos MediaCT 2012). This is likely to be influenced by the development of a wider range of games, many of which are designed to be appealing to target groups that deviate far beyond the traditional “profile” of a teenage male video gamer. Some recent evidence also indicates that choice of gaming device is relevant, as Pallesen et al. (2016) found that females are more likely than males to play games through social media.

### Strengths and Limitations

The findings from the present study have several important theoretical and potentially practical implications. Generally, the research literature in the field of problematic video gaming is too heavily focused on narrow subsets of the population, that is, people who are known to be problematic gamers or who represent subgroups considered more likely to develop such problems. Such convenience and strategic sampling is insufficient to understand the extent and characteristics of gamers in general. In contrast, our sample was representative of the general population. In order to deepen our understanding of and to refine our efforts to deal effectively with both video gaming and gambling, representative samples need be used from which generalizable results can be obtained. In addition to the large and representative sample, the validity of our conclusions is reinforced by the fact that the response rate was relatively high and that the distribution of male and female respondents was fairly even. Another asset of the current study is that it employed a longitudinal design that models the directions of relationships between variables. This allowed us to indicate which type of addictive behavior is predictive of the other, and how each behavior is related to potential confounding variables. The overall novelty of the field of video gaming research and the relative lack of studies that explicitly explore the association between video gaming and gambling make the current findings and research approach informative and relevant for future investigations.

In term of limitations, it should be noted that all data were self-reported. Such data are susceptible to biased self-descriptions, limitations in recollection and other sources of inaccurate reporting (Arnold and Feldman 1981). As the age of the current sample ranged from 16 to 74, it can be questioned whether this instrument, which was developed and validated for use on adolescents (Lemmens et al. 2009), was suitable for the current sample. However, studies have shown that the GASA has good psychometric properties when applied to adult samples (Andreassen et al. 2016; Festl et al. 2013).

One important methodological concern relates to the longitudinal design of the study, more specifically, the appropriateness of choosing two points of measurement separated by 2 years for exploring the relationship between the variables. It has been argued that due to the phenomenon of regression towards the mean, a minimum of three waves of measurement are needed to confidently draw causal inferences from longitudinal data (Segrin 2010). It should be noted, that several comparable studies in the field of video gaming and gambling have employed two points of measurement over even more limited periods (Lemmens et al. 2011a, b). One might expect that phenomenon to be flexible, innovative and ever changing as video gaming and gambling are more subject to change over brief periods of time. Therefore, there seems to be a high demand for studies that can detect transitions occurring within relatively short time periods. Another possible methodological limitation relates to the chosen method of analysis, as cross-lagged models have been criticized for presuming multiple assumptions that are unlikely to be met in reality (Segrin 2010). The majority of respondents in this study were neither problematic gamers nor problematic gamblers but the scales used in the present study represent continuous scores of problematic gaming and gambling. Thus, the present study should not be taken as categorical evidence that problem gamers develop into problem gamblers, but rather that the level of problem gaming is associated with a subsequent level of problem gambling, with the known caveat that much of the observed variation occurred on the lower end of both measures. Whether or not problem gamers as a category are at increased risk of developing subsequent problem gambling is a question that should be addressed in future research.

## Implications and Directions for Future Research

Because only a few studies have explicitly compared problematic video gaming and gambling, especially with representative samples and longitudinal designs, the relationship between these variables remains largely unclear. This highlights the point made by some authors that a focus solely on problematic video gaming or gambling might limit our understanding of these phenomena, as some research indicates that video gaming and gambling can yield some positive effects for its users as well (Granic et al. 2014). Broadening the interest in this manner might expand our understanding of the differences between normal and pathological involvement, and the causes and trajectories involved when these lines are crossed. Therefore, it would seem that more longitudinal studies based on representative samples are warranted.

One factor we did not explore was the influence of types of games played, which seems to be an important nuance, especially when considering male and female gambling preferences. In a similar vein, one might also suspect that the strong relationship between young age and video gaming and gambling will lessen with time. Such a trend would be expected due to the likely cohort-effects involved in usage of technological entertainment and developmental matureness (e.g., biological), but could be hastened by increased competence within older cohorts and improved efforts from developers to reach wider audiences. Given the massive public interest in, availability of, and increasing use of the Internet, it seems highly plausible that the groups of people who are involved in video gaming as well as the ways in which video games are played will continue to expand and diversify.

## Conclusion

The present study explored the prospective relationships between video gaming and gambling in a representative sample of the general Norwegian population using two waves of data collection spaced by an interval of 2 years. The results indicated that scores on the problem gaming scale predicted scores on the problem gambling scale, whereas the reverse relationship was not found. The scores on the two instruments predicted their respective scores on the same measure 2 years later, suggesting some consistency of measures of gaming and gambling problems. Given that both the variety and diversity of video gaming and gambling are rapidly developing, changing and expanding, one should continue to monitor the impact that involvement in either of the two behaviors has on the other, in both the short and the long term.
